# Simultaneous detection of lung fusions using a multiplex RT-PCR next generation sequencing-based approach: a multi-institutional research study

**DOI:** 10.1186/s12885-018-4736-4

**Published:** 2018-08-16

**Authors:** Cecily P. Vaughn, José Luis Costa, Harriet E. Feilotter, Rosella Petraroli, Varun Bagai, Anna Maria Rachiglio, Federica Zito Marino, Bastiaan Tops, Henriette M. Kurth, Kazuko Sakai, Andrea Mafficini, Roy R. L. Bastien, Anne Reiman, Delphine Le Corre, Alexander Boag, Susan Crocker, Michel Bihl, Astrid Hirschmann, Aldo Scarpa, José Carlos Machado, Hélène Blons, Orla Sheils, Kelli Bramlett, Marjolijn J. L. Ligtenberg, Ian A. Cree, Nicola Normanno, Kazuto Nishio, Pierre Laurent-Puig

**Affiliations:** 1 ARUP Institute for Clinical and Experimental Pathology, Salt Lake City, UT USA; 2 i3S - Instituto de Investigação e Inovação em Saúde, Universidade do Porto, Rua Alfredo Allen 208, 4200-135 Porto, Portugal; 3 IPATIMUP - Institute of Molecular Pathology and Immunology of the University of Porto, Rua Alfredo Allen 208, 4200-135 Porto, Portugal; 4 Medical Faculty of the University of Porto, Porto, Portugal; 5 Department of Pathology and Molecular Medicine, Queen’s University, Kingston, ON Canada; 6 Thermo Fisher Scientific, Austin, TX USA; 7 Laboratory of Pharmacogenomics, Centro di Ricerche Oncologiche di Mercogliano (CROM)-Istituto Nazionale Tumori “Fondazione G. Pascale”-IRCCS, Naples, Italy; 8 Pathology Unit, Istituto Nazionale Tumori “Fondazione G. Pascale”-IRCCS, Naples, Italy; 9 Department of Pathology, Radboud University Medical Center, Nijmegen, the Netherlands; 10 Viollier AG, Department of Genetics/Molecular Biology, Basel, Switzerland; 11 Department of Genome Biology, Kinki University Faculty of Medicine, Osaka, Japan; 12 ARC-NET: Centre for Applied Research on Cancer, Department of Pathology and Diagnostic, University and Hospital Trust of Verona, Verona, Italy; 13 Department of Pathology, University Hospitals Coventry and Warwickshire, Walsgrave, Coventry, UK; 14 University Paris Descartes, Paris, France; 15 Biology Department, Assistance Publique-Hôpitaux de Paris, European Georges Pompidou Hospital, Paris, France; 16 Institute of Pathology, University Hospital Basel, Basel, Switzerland; 17 Luzerner Kantonsspital, Luzern, Switzerland; 18 Trinity Translational Medicine Institute (TTMI), Trinity College Dublin, Dublin, Ireland; 19 Department of Human Genetics, Radboud University Medical Center, Nijmegen, the Netherlands; 20 Cell Biology and Biotherapy Unit, Istituto Nazionale Tumori “Fondazione G. Pascale”-IRCCS, Naples, Italy; 21 Centre for Sport, Exercise and Life Sciences, Coventry University, Coventry, UK

**Keywords:** Gene fusions, Detection, Biomarker, Lung cancer, Next-generation sequencing, FFPE

## Abstract

**Background:**

Gene fusion events resulting from chromosomal rearrangements play an important role in initiation of lung adenocarcinoma. The recent association of four oncogenic driver genes, *ALK*, *ROS1*, *RET*, and *NTRK1*, as lung tumor predictive biomarkers has increased the need for development of up-to-date technologies for detection of these biomarkers in limited amounts of material.

**Methods:**

We describe here a multi-institutional study using the Ion *AmpliSeq*™ RNA Fusion Lung Cancer Research Panel to interrogate previously characterized lung tumor samples.

**Results:**

Reproducibility between laboratories using diluted fusion-positive cell lines was 100%. A cohort of lung clinical research samples from different origins (tissue biopsies, tissue resections, lymph nodes and pleural fluid samples) were used to evaluate the panel. We observed 97% concordance for *ALK* (28/30 positive; 71/70 negative samples), 95% for *ROS1* (3/4 positive; 19/18 negative samples), and 93% for *RET* (2/1 positive; 13/14 negative samples) between the AmpliSeq assay and other methodologies.

**Conclusion:**

This methodology enables simultaneous detection of multiple *ALK*, *ROS1*, *RET*, and *NTRK1* gene fusion transcripts in a single panel, enhanced by an integrated analysis solution. The assay performs well on limited amounts of input RNA (10 ng) and offers an integrated single assay solution for detection of actionable fusions in lung adenocarcinoma, with potential savings in both cost and turn-around-time compared to the combination of all four assays by other methods.

**Electronic supplementary material:**

The online version of this article (10.1186/s12885-018-4736-4) contains supplementary material, which is available to authorized users.

## Background

Non-small cell lung carcinoma (NSCLC) has been categorized into several distinct entities by molecular characterization of genetic alterations occurring during epithelial cell transformation. These alterations lead mainly to the activation of oncogenes such as *EGFR*, *KRAS*, *NRAS*, *BRAF* or *ERBB2,* [1–3] which occur through point mutations, small deletions or insertions and, more rarely, amplifications. Several other key drivers have been implicated in lung cancer carcinogenesis through other mechanisms. Indeed, chromosomal rearrangements involving the tyrosine kinase receptor genes *ALK*, [4] *ROS1*, [5] *RET*, [6–8] and *NTRK1*, [9] have been more recently described, extending the repertoire of molecular alterations found in NSCLC. These fusion events, involving a variety of partner genes, result in the formation of chimeric fusion kinases capable of oncogenic transformation and induction of oncogene dependency within the neoplastic cells. The prevalence of each of these chromosomal rearrangements individually is 1–7% in NSCLC [4, 6, 10, 11], and altogether can be identified in approximately 5–9% of NSCLC [7, 12, 13].

The development of drugs that specifically target fusion proteins encoded by these rearrangements [9, 11, 14] has driven the need for systematic sensitive assays to detect them. Lung cancer fusions have traditionally been detected using FISH, IHC, or RT-PCR. While FISH is considered the gold standard, especially for ALK testing due to the availability of an FDA-approved ALK FISH assay, FISH analysis for multiple targets per sample can be costly. The massively parallel nature of next generation sequencing (NGS) allows a rapid characterization of point mutations, small insertions and deletions. Additionally, NGS can be used for the detection of chromosome rearrangements in a large set of genes by targeted sequencing of the fusion junctions or by paired-end mapping methods. In this study we validated a new library kit, the Ion AmpliSeq™ RNA Fusion Lung Cancer Research Panel, for characterization of the most frequent chromosome rearrangements in lung adenocarcinoma by NGS. This library kit is based on the high-multiplexing capabilities of PCR and focuses on the identification of 72 different transcripts. We report the sensitivity and specificity of this assay for the detection of gene fusions implicated in NSCLC.

## Methods

### Samples

A total of 138 clinical research samples previously tested for *ALK*, *ROS1*, and/or *RET* rearrangements were collected from 10 participating laboratories. All clinical research samples were studied in the laboratory of origin. All samples were from resections or biopsies that had been formalin-fixed and paraffin-embedded (FFPE), with the exception of three fresh frozen samples (one resection and two pleural effusions). These included 128 samples previously tested for *ALK* rearrangements by fluorescence in situ hybridization (FISH). Sixty-five of these samples had also been tested for *ALK* rearrangements by another method: immunohistochemistry (IHC), reverse transcription (RT)-PCR, and/or mass spectrometry (performed on the MassARRAY System from Agena Bioscience, San Diego, CA). Categorization of the *ALK*-tested samples as positive, negative or inconclusive was determined by the FISH results, as this methodology is considered the gold standard for *ALK* testing. For those samples previously tested by multiple methods, any discrepancies in results between the methodologies were noted. Thirteen of the *ALK* samples had also been tested for *ROS1* and/or *RET* rearrangements. An additional 10 clinical research samples previously tested for *ROS1* and/or *RET*, but for which *ALK* testing results were unavailable, were also included in this study. Categorization of the *ROS1* and *RET* samples was based on the results from any available method, including FISH, IHC, RT-PCR and/or mass spectrometry, since there is not an established gold-standard for detection of these rearrangements.

RNA was extracted from each of the clinical research samples by the participating laboratories using their respective standard extraction procedures. Six of the ten laboratories used the RecoverAll Total Nucleic Acid Isolation Kit for FFPE (Thermo Fisher Scientific, Waltham, MA); remaining labs used the Qiagen RNeasy FFPE Kit (Qiagen, Hilden, Germany), the Qiagen AllPrep DNA/RNA FFPE Kit, or the Maxwell LEV RNA FFPE Purification Kit (Promega, Madison, WI). RNA was quantified using the Qubit RNA assay kits (Thermo Fisher) at eight of the laboratories; Quant-iT RiboGreen RNA Assay Kit (Thermo Fisher) and the Nanodrop 2000 instrument (Thermo Scientific) were also used for quantification.

In addition to the clinical research samples, a cocktail of RNA isolated from the *ALK* fusion-positive H2228 (ATCC CRL-5935), *ROS1* fusion-positive HCC-78 (DSMZ ACC 563), and *RET* fusion-positive LC-2/ad (ECACC LC-2/ad) cell lines was prepared by Thermo Fisher Scientific and supplied to each of the participating laboratories. Select laboratories also prepared and tested RNA isolated from FFPE versions of these cell lines and RNA isolated from the *ALK* fusion-positive cell line H3122 (ECACC NCI-H322) and the *NTRK1* fusion-positive cell line KM-12.

### Ion *AmpliSeq* RNA fusion lung Cancer research panel design

Primers spanning 72 fusions (37 *ALK*, 9 *RET*, 15 *ROS1*, and 11 *NTRK1*) were designed by a research team at Thermo Fisher. These primers were designed to span all previously described fusions, at the time of development, for *ALK*, *ROS1*, *RET*, and *NTRK1* in lung cancers. Sources used for the curation of known fusions included the COSMIC and NCBI databases, and review of current medical literature. Targeted fusion genes are shown in Table 1. The multiplex primer mix also included primers for the amplification of five housekeeping genes: *HMBS*, *ITGB7*, *LMNA*, *MYC*, and *TBP*.

**Table 1:** Targeted Partners for *ALK*, *RET*, *ROS1*, and *NTRK1*

ALK	RET	ROS1	NTRK1
EML4	KIF5B	CD74	CEL
KIF5B	CCDC6	SDC4	NFASC
KLC1	CUX1	SLC34A2	IRF2BP2
HIP1		EZR	TFG
TPR		TPM3	SQSTM1
		LRIG3	SSBP2
		GOPC	CD74
			DYNC2H1
			MPRIP

Additionally, primers designed to amplify 5′ and 3′ regions of *ALK*, *ROS1*, *RET*, and *NTRK1* were included in the primer mix. Amplification of these regions for each gene of interest allowed for the comparison of expression levels between the 3′ end of the gene, which is part of the resulting fusion, and the non-involved 5′end of the gene. A list of all targets in the multiplex PCR – including targeted fusions (genes and exons), expression control genes, and 3′and 5′regions – is available in Additional file 1: Table S1.

### Detection of fusions

A minimum of 10 ng of total RNA was reverse transcribed using the SuperScript VILO cDNA Synthesis Kit followed by library generation using the Ion AmpliSeq Library Kit 2.0 and the Ion AmpliSeq RNA Fusion Lung Cancer Research Panel (hereafter, AmpliSeq Fusion Lung Panel). Barcodes were utilized during library generation using the Ion Xpress Barcode Adapters. Libraries were quantified using the Qubit DNA assay, the 2100 BioAnalyzer (Agilent Technologies, Santa Clara, CA) or the Ion Library Quantitation Kit, then pooled in equimolar concentrations for sequencing. Eight to sixteen libraries were multiplexed and templated using the Ion OneTouch2 System with the Ion PGM Template OT2 200 Kit. Libraries were sequenced using the Ion PGM Sequencing 200 v2 kit on an Ion 316 v2 or 318 v2 chip on the Ion PGM instrument. (All reagents and instrumentation above are from Thermo Fisher Scientific, with the exception of the BioAnalyzer.) Typically, eight samples were sequenced per 316 chip and sixteen samples per 318 chip.

After sequencing, unaligned BAM files were transferred to the Ion Reporter Software 4.2 and analyzed using the AmpliSeq Lung Fusion single sample workflow. This workflow utilizes a BED file comprised of chimeric sequences for targeted fusion transcripts along with sequences for the expression control genes and the 3′and 5′regions of *ALK*, *ROS1*, *RET*, and *NTRK1*. The alignment consists of three main steps. In the first step, the aligner requires that the reads align end to end (i.e, reads that are trimmed, or soft clipped, at the ends are not allowed). Each read is then aligned to the best primary alignment and filtering criteria are applied. Alignments to the fusion targets are counted only if the read overlaps at least 70% of the expected fusion insert with high local alignment score. Alignments to the imbalance and control targets are counted if the read overlaps at least 50%. In the second step, all unaligned reads, and reads that aligned but were filtered out, are split into two fragments. These fragmented reads are then re-aligned to the same reference file. Trimming of the reads is allowed in this step and all the alignments of every read (not just the primary alignments) are kept in the alignments files. This step helps recover more counts for the targets in the reference file and also finds any non-targeted fusion isoforms that are not present in the original list of targets. A novel fusion isoform involving existing primers is reported in the output if there is evidence from at least 100 different pairs of fragments. Lastly, counts from steps one and two are aggregated and all the fusion targets that have counts higher than the threshold are reported as “fusion present.” The algorithm generates a 3′/5′expression imbalance metric for each of the driver genes based on the individual counts of the 5′assay and 3′assay. It is calculated by subtracting the count of 5′reads from the count of 3′reads, and dividing the result by the sum of counts of all control targets. This metric can be used to confirm the detection of a known fusion or to predict a fusion in the sample that is not covered by the isoforms in the panel.

## Results

### Cell lines

Using the cocktail of RNA from the *ALK*, *ROS1*, and *RET* fusion-positive cell lines, each of the ten participating laboratories successfully detected all three rearrangements using the AmpliSeq Fusion Lung Panel assay (see Table 2). The fusions detected corresponded to the rearrangements previously described for these cell lines [11, 15–18] (see Table 3). The expected rearrangements were also detected from RNA isolated from the FFPE cell blocks of the same cell lines and from the *ALK*-positive H3122 cell line [11] (Tables 2 and 3). In the KM12 cell line [19], primers for the specific NTRK fusion were not included in the assay design, but the rearrangement was detected by a positive 3′/5′imbalance result of 0.076, above the cut-off of 0.025 (Table 2).

**Table 2:** Control Sample Results Across Participating Laboratories

Laboratory	Sample	ALK fusion	ROS1 fusion	RET fusion	NTRK1 fusion	ALK 3′/5′ imbalance	ROS1 3′/5′ imbalance	RET 3′/5′ imbalance	NTRK 3′/5′ imbalance
INSERM	Cell Line RNA cocktail^a^	Detected	Detected	Detected	n/a	0.0044	0.3031	0.0603	n/a
Kinki	Cell Line RNA cocktail^a^	Detected	Detected	Detected	n/a	0.0405	0.4648	0.0434	n/a
ARUP	Cell Line RNA cocktail^a^	Detected	Detected	Detected	n/a	0.0172	0.6141	0.0421	n/a
ARC-Net	Cell Line RNA cocktail^a^	Detected	Detected	Detected	n/a	0.0086	0.3458	0.0353	n/a
Viollier	Cell Line RNA cocktail^a^	Detected	Detected	Detected	n/a	0.043	0.3398	0.046	n/a
Radboudumc	Cell Line RNA cocktail^a^	Detected	Detected	Detected	n/a	0.0414	0.2502	0.0856	n/a
CROM	Cell Line RNA cocktail^a^	Detected	Detected	Detected	n/a	0.012	0.054	0.0813	n/a
IPATIMUP	Cell Line RNA cocktail^a^	Detected	Detected	Detected	n/a	0.0238	0.2877	0.0539	n/a
Warwick	Cell Line RNA cocktail^a^	Detected	Detected	Detected	n/a	0.0064	0.3	0.055	n/a
Queen’s	Cell Line RNA cocktail^a^	Detected	Detected	Detected	n/a	0.0123	0.4158	0.0478	n/a
ARUP	FFPE cell block H2228	Detected	n/a	n/a	n/a	0.0312	n/a	n/a	n/a
ARUP	FFPE cell block HCC78	n/a	Detected	n/a	n/a	n/a	3.8313	n/a	n/a
ARUP	FFPE cell block LC-2/ad	n/a	n/a	Detected	n/a	n/a	n/a	0.2521	n/a
Kinki	Fresh cells H3122	Detected	n/a	n/a	n/a	0.3165	n/a	n/a	n/a
ARUP	FFPE cell block KM-12	n/a	n/a	n/a	Not covered	n/a	n/a	n/a	0.076

^a^A mixture of RNA from three cell lines (H2228, HCC78, and LC-2/ad)

**Table 3:** Cell Line Fusions

Cell line	Expected fusion (s)	Detected fusion (s)
H2228	EML4(6a,6b)-ALK(20)	EML4(6a,6b)-ALK(20)
HCC78	SLC34A2(4)-ROS1(32,34)	SLC34A2(4)-ROS1(32,34,35,36)
LC-2/ad	CCDC6(1)-RET(12)	CCDC6(1)-RET(12)
H3122	EML4(13)-ALK(20)	EML4(13)-ALK(20)
KM-12	TPM3(7)-NTRK1(8)	not covered

### *ALK* clinical samples

Of the 138 clinical research samples tested, 117 (84.5%) passed the QC requirement of a minimum of 20,000 total reads. One hundred of these samples had previously been tested for *ALK* rearrangements by FISH with conclusive results; AmpliSeq Fusion Lung Panel results were 97% concordant (97/100 samples) with FISH analysis (see Table 4**)**. *ALK* rearrangements were detected in 28/30 *ALK* FISH-positive samples, with a sensitivity of 93.3%; exact fusions were identified in 24 of the samples and an additional 4 samples showed evidence of rearrangement using the 3′/5′imbalance calculation. In samples negative for *ALK* rearrangements by FISH, 69 of 70 samples were also negative by the AmpliSeq assay, thus resulting in a specificity of 98.6%. Details on all *ALK* clinical research samples are shown in Additional file 1: Table S2.

**Table 4:** Concordance Between FISH and AmpliSeq for Detection of *ALK* Fusions

	FISH +	FISH -
AmpliSeq +	28	1
AmpliSeq -	2	69

Closer analysis of the discordant results (see Table 5), revealed that two of the FISH-positive *ALK* samples without a detected fusion showed 10% rearranged cells (below the usual cut-off of 15%). One of these samples showed only weak staining by IHC, and subsequent re-testing by the AmpliSeq Fusion Lung Panel gave a positive result of a fusion of *HIP1*-*ALK* with 30 reads. Five of the remaining seven discordant *ALK* FISH-positive samples showed an atypical FISH result of one red signal, rather than the split red-green signal; of these, two were IHC negative for ALK and one was positive by IHC originally, but negative upon repeat testing. The remaining two FISH-positive, AmpliSeq fusion-negative samples showed typical FISH results with 19% and 20% rearranged cells, respectively, and both were positive for ALK IHC staining.

**Table 5:** Discordant ALK clinical samples

Sample	Laboratory	FISH (rearranged cells)	AmpliSeq result (n° reads)	3′-5′ imbalance	IHC results
26	Viollier	Positive (19%)	Negative (136146)	0,0099	Positive
30	CROM	Positive (20%)	Negative (166403)	0,0008	2+
107	Queen’s	Negative	EML4(6a)-ALK(20) (1835387)	0,3179	Positive

One discordant sample was negative by FISH and positive by AmpliSeq. This sample showed an *EML4*- *ALK* fusion with 6137 fusion reads, and the 3′/5′imbalance in this sample also showed a positive result. Additionally, this sample had previously tested positive for ALK protein expression by IHC.

### *ROS1* and *RET* clinical samples

Panel results for *ROS1* and *RET* were concordant in 21/22 samples (95.5%) for *ROS1* and 14/15 (93.3%) for *RET* as compared to previous testing using available methods, including FISH, IHC, RT-PCR, and mass spectrometry. The AmpliSeq assay detected the appropriate fusions in 3/4 *ROS1*-positive samples and in 1/1 *RET*-positive sample. Samples previously determined to be negative for *ROS1* using other methodologies (18 samples) were all negative using the AmpliSeq Fusion Lung Panel. In samples previously determined to be negative for *RET* fusions, 13 of 14 were negative by the AmpliSeq panel, and one showed a positive imbalance result of 0.197, in the absence of a detected fusion isoform (Additional file 1: Table S3). This sample was subsequently tested using the *RET* FISH break-apart probe and also re-run using the AmpliSeq panel; both results were negative.

### Detection and confirmation of additional fusions

Testing of the clinical research samples yielded the detection of *ROS1* fusions in two samples which were *ALK*-negative by FISH (Samples 63 and 67, Additional file 1: Table S2). In both cases, the *CD74*-*ROS1* fusion was detected and subsequent testing using a TaqMan assay with primers specific for the detected fusion confirmed the results. Prior to this study, neither of these samples had been tested for *ROS1* rearrangements.

*RET* fusions were detected in three of the *ALK*-negative samples (Samples 48, 55, and 98, Additional file 1: Table S2). In Sample 98, the *KIF5B*-*RET* fusion was detected and subsequently confirmed by a TaqMan assay. Samples 48 and 55 showed positive *RET* 3′/5′imbalance results of 0.041 and 0.271, respectively. Subsequent FISH analysis of Sample 55 showed 10% split signals in the tissue area used for extraction of RNA for the AmpliSeq Fusion Lung Panel assay. Additional material for confirmatory testing was not available for Sample 48.

Lastly, a *ROS1* fusion was detected with 83 fusion reads in one of the *ALK* FISH-positive samples (Sample 8, Additional file 1: Table S2). The presence of two fusion events is unlikely, and subsequent testing by RT-PCR did not confirm the presence of this fusion.

### Samples with low Total reads

Upon initial evaluation of the 138 clinical research samples, samples below the QC cut-off of 20,000 were repeated with either 30 PCR cycles or simply re-pooling prior to bead templating. Five of ten samples successfully repeated, and those samples are included in the data above. In the other five samples and in samples for which repeat testing was not possible, reasons for failure included insufficient RNA quantity (< 10 ng), degraded RNA, and improper pooling of libraries.

## Discussion

The advent of therapies targeting the fusion proteins arising from *ALK*, *ROS1*, and *RET* gene fusions makes the routine detection of these events important in patients with lung adenocarcinoma. We have described here an international, multi-institutional study using a multiplex RT-PCR next generation sequencing-based method that enables simultaneous detection of *ALK*, *RET*, *ROS1*, and *NTRK1* gene fusion transcripts in a single assay. The simultaneous detection of these fusions has important implications for turn-around-time and cost. Further, it can be performed with very little input RNA. This is particularly attractive for an assay targeted at lung cancers, as these samples are often biopsies with limited available tissue. Lung cancer fusions have traditionally been detected using FISH, IHC, or RT-PCR. While FISH is considered the gold standard, especially for *ALK* testing due to the availability of an FDA-approved *ALK* FISH assay, FISH analysis for multiple targets per sample can be costly. Often these analyses are done in step-wise fashion, which can reduce the overall cost of performing multiple FISH assays, but potentially extend the time needed to rule out all relevant gene rearrangements. Immunohistochemistry staining offers a cheaper alternative; however, this methodology is subjective, sometimes making interpretation difficult. [20] RT-PCR, on the other hand, can offer precise detection of fusions, including identification of both partner genes and the exons involved. The main limitation of traditional RT-PCR is that it typically focuses on only the most common fusion events and is thus limited in detecting rare exon combinations. [21]

In contrast to FISH or IHC, the detection of *ALK*, *ROS1*, *RET*, and *NTRK1* fusions are combined in a single assay with the AmpliSeq design. From the 70 clinical research samples that previously had been determined to be *ALK*-negative by FISH, we detected two *ROS1* fusions and three *RET* fusions. Both of the *ROS1* fusions and two of *RET* fusions were confirmed to be positive by orthogonal methods; tissue for additional testing was not available for the third *RET*-positive sample. Further, the detection of fusions by NGS offers a timely methodology that can also be designed to accommodate the simultaneous detection of point mutations and insertions and deletions in the DNA of relevant genes in a single assay. Analysis of these types of mutations, particularly in *EGFR* and *KRAS*, is typically part of the work-up of lung adenocarcinoma patients. Methods to detect both DNA mutations and fusion events in a timely manner are particularly important in these patients due to the aggressive nature of the disease. While combined analysis of DNA and RNA was not the focus of this study, it is currently being performed by many of the institutions that participated in this study.

The methodology described in this paper relies on RT-PCR for the initial amplification of fusion events; however, the design of this assay circumvents a limitation of traditional RT-PCR. The AmpliSeq Fusion Lung Panel assay includes multiplexing of primers for 72 different fusion combinations and thus is not limited to only the most common fusions. A second limitation of traditional RT-PCR is that one must have previous knowledge of all possible relevant fusions. The AmpliSeq assay addresses this issue in two ways. First, during the analysis of the sequenced reads, all reads that are initially unaligned to the reference sequence are split in half and allowed to re-align. This step fosters the detection of novel fusions involving existing primers. Secondly, the assay includes a method for detection fusions involving unknown partners using the 3′/5′imbalance calculation. This step analyzes the expression levels of the 3′ and 5′ends of each driver gene. For genes involved in a fusion event, the 3′ end of the gene is now under different regulatory control and shows overexpression relative to the 5′end of the gene. Another recently described methodology using NanoString technology also exploits this phenomenon of 3′overexpression. [22] That study found that evaluation of the imbalance between 3′and 5′expression works relatively well for *ALK* and *RET*, which are normally not expressed in lung tissue, but that this calculation was more difficult for *ROS1* as this gene is normally expressed at high levels. Given that a positive imbalance result is suggestive of a fusion event, but alone does not identify an exact fusion, our suggestion for the AmpliSeq assay is to use the imbalance calculation as a method for identifying possible fusions that should be followed up with orthogonal testing methods if desired.

Further analysis of discordant samples within our study found that some samples had either low levels of rearranged cells by FISH or discordant results between FISH and IHC. One of the samples for which FISH testing showed 10% rearranged cells, was positive for a *HIP1*-*ALK* fusion upon repeat testing with the AmpliSeq assay. The repeat result had fusion reads falling just above the cut-off, while the initial negative result did identify the same fusion but with a number of reads falling below the cut-off, indicating the sample was likely approaching the limit of detection for the assay. Discordance between *ALK* FISH and other methods has been noted previously [23–25] and brings up the question of a true “gold standard.” Three of the *ALK* FISH-positive samples for which the AmpliSeq assay was negative, were also negative by IHC. Additionally, we found that five of the discordant samples displayed single red signals by FISH. This phenomenon of a single red signal represents a likely deletion of the 5′end of *ALK* and is not unusual for this structural variant; however, previous studies have also shown a similar discordance between *ALK* FISH-positive results displaying a deletion of the 5′*ALK* probe and IHC [24] or PCR. [26] The exact nature of these fusion events may be of interest for future studies. We also observed discordant results for one of the *ALK* FISH-negative samples. In this case, the AmpliSeq assay identified an *EML4*-*ALK* fusion with a high number of reads and the sample was also positive by IHC. While this sample was officially classified as an AmpliSeq “false positive,” it likely represents a true positive in which FISH testing failed to detect the fusion.

A recent study using the AmpliSeq method for fusion detection reported 100% concordance between this and other methodologies. [27] It is unknown, but probable, that the testing for this study was performed at a single institution. The difference between a single or limited institution study and a larger study (in this case, ten institutions) may explain the difference in concordance results between the Pfarr study [27] and the study described here. The international, multi-institutional nature of this study presented many challenges. Scoring criteria between laboratories often varies even for well-established reference methods, e.g., some samples in this study were deemed FISH-positive, yet fell below the cut-off of 15% used by other institutions. A lack of concordance between multiple institutions for detection of *ALK* rearrangements has been previously observed, [20, 28] and this phenomenon may have contributed to the lower concordance of compared methods in this study. A further challenge of the multi-institutional study included a lack of material for follow up on discrepant samples, as the samples were not only from the participating institutions but in some cases were from additional laboratory partners. However, we believe that the advantages of this multi-institutional study far outweigh the disadvantages. Reproducibility across different laboratories using cell line mixtures was 100%, despite potential differences in laboratory practices and personnel. Additionally, an international, multi-institutional study such as this allows for the inclusion of more varied samples and more fully explores the performance of the assay.

## Conclusion

The RT-PCR NGS assay described here offers many advantages for laboratory testing in lung adenocarcinoma samples. This methodology allows detection of multiple fusions in a single assay and can easily also be multiplexed with detection of point mutations and small insertions and deletions in genes such as KRAS and EGFR that are also important in the work up of these patients. The single-assay format potentially allows for faster turn-around-time and lower cost than doing the assays separately. Further, the small amount of input RNA required is very advantageous for these samples. However, the AmpliSeq assay primarily targets known fusions. Inclusion of the 3′/5′imbalance calculation aims to address this limitation, but could likely benefit from further refinement of cut-offs values as more data is generated by this assay. Lastly, efforts to periodically update the primer pool as additional partner genes for *ALK*, *ROS1*, *RET*, and *NTRK1* fusions are identified would aid in the continuing utility of this assay.

## Additional file

Additional file 1: **Table S1.** RT-PCR Targets within the Ion AmpliSeq™ Lung Fusion Research Panel. A list of all targets in the multiplex PCR studied – including targeted fusions (genes and exons), expression control genes, and 3′and 5′regions. Table S2. *ALK* Clinical Samples. Table depicting details on all *ALK* clinical research samples analyzed in the study. Table S3. *ROS1* and *RET* clinical samples. Table depicting details on all *ROS1* and *RET* clinical research samples analyzed in the study. (DOCX 57 kb)

## Abbreviations

FFPE: Formalin-fixed paraffin-embedded
FISH: Fluorescence in situ hybridization
IHC: Immunohistochemistry
NGS: Next generation sequencing
NSCLC: Non-small cell lung cancer
RT-PCR: Reverse transcriptase-polymerase chain reaction
