# Supplementary material for: Simultaneous detection of lung fusions using a multiplex RT-PCR next generation sequencing-based approach: a multi-institutional research study
Source: BMC Cancer. 2018 Aug 16;18:828. doi: 10.1186/s12885-018-4736-4 (PMC6097211; doi:10.1186/s12885-018-4736-4)
Supplement: Supplementary file 1 — Table S1. RT-PCR Targets within the Ion AmpliSeq™ Lung Fusion Research Panel. A list of all targets in the multiplex PCR studied – including targeted fusions (genes and exons), expression control genes, and 3′and 5′regions. Table S2. ALK Clinical Samples. Table depicting details on all ALK clinical research samples analyzed in the study. Table S3. ROS1 and RET clinical samples. Table depicting details on all ROS1 and RET clinical research samples analyzed in the study. (DOCX 57 kb) [file 12885_2018_4736_MOESM1_ESM.docx]

| **Table S1. RT-PCR Targets within the Ion AmpliSeq™ Lung Fusion Research Panel** | | |
| --- | --- | --- |
| **Targeted genes (exons)** | **Reference** | **Assay type** |
| EML4(6)-ALK(19) | COSF1296 | Fusion |
| EML4(6)-ALK(19) isoform | COSF1296.1 | Fusion |
| EML4(2)-ALK(20) | COSF479 | Fusion |
| EML4(2)-ALK(20) isoform | COSF479.1 | Fusion |
| EML4(15)-ALK(20) | COSF413 | Fusion |
| EML4(17)-ALK(20) | COSF1367 | Fusion |
| EML4(17)-ALK(20) isoform | COSF1367.1 | Fusion |
| EML4(17)-ALK(20) | COSF1366 | Fusion |
| EML4(17)-ALK(20) isoform | COSF1366.1 | Fusion |
| EML4(14)-ALK(20) | COSF477 | Fusion |
| EML4(14)-ALK(20) isoform | COSF477.1 | Fusion |
| EML4(14)-ALK(20) | COSF1064 | Fusion |
| EML4(13)-ALK(20) | COSF1062 | Fusion |
| EML4(13)-ALK(20) isoform | COSF1062.1 | Fusion |
| EML4(13)-ALK(20) | COSF408 | Fusion |
| EML4(13)-ALK(20) | AB462411 | Fusion |
| EML4(18)-ALK(20) | COSF487 | Fusion |
| EML4(18)-ALK(20) isoform | COSF487.1 | Fusion |
| EML4(2)-ALK(20) | COSF478 | Fusion |
| EML4(2)-ALK(20) isoform | COSF478.1 | Fusion |
| EML4(20)-ALK(20) | COSF409 | Fusion |
| EML4(20)-ALK(20) isoform | COSF409.1 | Fusion |
| EML4(20)-ALK(20) | COSF730 | Fusion |
| EML4(20)-ALK(20) isoform | COSF730.1 | Fusion |
| EML4(6a)-ALK(20) | COSF411 | Fusion |
| EML4(6a)-ALK(20) | AB374361 | Fusion |
| EML4(6b)-ALK(20) | COSF412 | Fusion |
| EML4(6b)-ALK(20) isoform | COSF412.1 | Fusion |
| EML4(6b)-ALK(20) | AB374362 | Fusion |
| KIF5B(15)-ALK(20) | COSF1381 | Fusion |
| KIF5B(15)-ALK(20) | COSF1060 | Fusion |
| KIF5B(17)-ALK(20) | COSF1257 | Fusion |
| KIF5B(24)-ALK(20) | COSF1058 | Fusion |
| KLC1(9)-ALK(20) | COSF1276 | Fusion |
| HIP1(28)-ALK(20) | ENST00000336926/ENST00000389048 | Fusion |
| HIP1(21)-ALK(20) | ENST00000336926/ENST00000389048 | Fusion |
| TPR(15)-ALK(20) | ENST00000367478/ENST00000389048 | Fusion |
| KIF5B(15)-RET(11) | COSF1255 | Fusion |
| KIF5B(24)-RET(11) | COSF1262 | Fusion |
| KIF5B(24)-RET(8) | COSF1236 | Fusion |
| CCDC6(1)-RET(12) | COSF1271 | Fusion |
| KIF5B(15)-RET(12) | COSF1232 | Fusion |
| KIF5B(16)-RET(12) | COSF1230 | Fusion |
| KIF5B(22)-RET(12) | COSF1253 | Fusion |
| KIF5B(23)-RET(12) | COSF1234 | Fusion |
| CUX1(10)-RET(12) | ENST00000292538/ENST00000355710 | Fusion |
| CD74(6)-ROS1(32) | COSF1202 | Fusion |
| CD74(6)-ROS1(34) | COSF1200 | Fusion |
| SDC4(2)-ROS1(32) | COSF1265 | Fusion |
| SDC4(4)-ROS1(32) | COSF1278 | Fusion |
| SDC4(2)-ROS1(34) | ENST00000372733/ENST00000368508 | Fusion |
| SDC4(4)-ROS1(34) | COSF1280 | Fusion |
| SLC34A2(13)-ROS1(32) | COSF1259 | Fusion |
| SLC34A2(4)-ROS1(32) | COSF1197 | Fusion |
| SLC34A2(4)-ROS1(34) | COSF1198 | Fusion |
| SLC34A2(13)-ROS1(34) | COSF1261 | Fusion |
| EZR(10)-ROS1(34) | COSF1267 | Fusion |
| TPM3(7)-ROS1(35) | COSF1273 | Fusion |
| LRIG3(16)-ROS1(35) | COSF1269 | Fusion |
| GOPC(8)-ROS1(35) | COSF1139 | Fusion |
| GOPC(4)-ROS1(36) | COSF1188 | Fusion |
| CEL(7)-NTRK1(7) | ENST00000372080/ENST00000392302 | Fusion |
| NFASC(18)-NTRK1(10) | ENST00000360049/ENST00000392302 | Fusion |
| IRF2BP2(1)-NTRK1(10) | ENST00000366609/ENST00000392302 | Fusion |
| TFG(5)-NTRK1(10) | ENST00000240851/ENST00000392302 | Fusion |
| SQSTM1(5)-NTRK1(10) | ENST00000389805/ENST00000392302 | Fusion |
| SSBP2(12)-NTRK1(12) | ENST00000320672/ENST00000392302 | Fusion |
| NTRK1(17)-DYNC2H1(86) | ENST00000392302/ENST00000375735 | Fusion |
| CD74(3)-NTRK1(13) | ENST00000353334/ENST00000524377 | Fusion |
| MPRIP(14)-NTRK1(13) | ENST00000395811/ENST00000524377 | Fusion |
| MPRIP(18)-NTRK1(13) | ENST00000395811/ENST00000524377 | Fusion |
| MPRIP(21)-NTRK1(13) | ENST00000395811/ENST00000524377 | Fusion |
| HMBS | ENST00000537841 | Expression Control |
| TBP | ENST00000392092 | Expression Control |
| ITGB7 | ENST00000267082 | Expression Control |
| MYC | ENST00000377970 | Expression Control |
| LMNA | ENST00000368300 | Expression Control |
| ALK 5' region | ENST00000389048 | 3'/5' Imbalance |
| RET 5' region | ENST00000340058 | 3'/5' Imbalance |
| ROS1 5' region | ENST00000368508 | 3'/5' Imbalance |
| NTRK1 5' region | ENST00000392302 | 3'/5' Imbalance |
| ALK 3' region | ENST00000389048 | 3'/5' Imbalance |
| RET 3' region | ENST00000340058 | 3'/5' Imbalance |
| ROS1 3' region | ENST00000368508 | 3'/5' Imbalance |
| NTRK1 3' region | ENST00000392302 | 3'/5' Imbalance |

| **Table S2. *ALK* Clinical Samples** | | | | | | | | |
| --- | --- | --- | --- | --- | --- | --- | --- | --- |
| **Sample** | **Laboratory** | **Sample type** | **Source** | **FISH result** | **AmpliSeq result** | **Detected fusion (exon)** | **3'/5' imbalance** | **Additional ALK testing (result)** |
| *Positive by ALK FISH* | | |  |  |  |  |  |  |
| 1 | INSERM | FFPE | Lung biopsy | Positive | Positive | EML4(13)-ALK(20) | 0.0763 | IHC (positive), RT-PCR (positive) |
| 2 | INSERM | FFPE | Lung biopsy | Positive | Positive | EML4(20)-ALK(20) | 0.1089 | IHC (positive), RT-PCR (positive) |
| 3 | INSERM | FFPE | Lung biopsy | Positive | Positive | EML4(6a)-ALK(20), EML4(6b)-ALK(20) | 0.0199 | IHC (positive), RT-PCR (positive) |
| 4 | INSERM | FFPE | Lung biopsy | Positive | Positive | EML4(20)-ALK(20) | 0.0170 | IHC (positive), RT-PCR (positive) |
| 5 | INSERM | FFPE | Lung biopsy | Positive | Positive | EML4(6a)-ALK(20), EML4(6b)-ALK(20) | 0.0277 | IHC (positive), RT-PCR (negative) |
| 6 | INSERM | FFPE | Lung biopsy | Positive | Positive | EML4(13)-ALK(20), EML4(20)-ALK(20) | -0.0074 | IHC (positive), RT-PCR (positive) |
| 7 | INSERM | FFPE | Lung biopsy | Positive | Positive | EML4(13)-ALK(20) | 0.0206 | IHC (positive), RT-PCR (positive) |
| 8 | INSERM | FFPE | Lung biopsy | Positive | Positive | EML4(6a)-ALK(20), EML4(6b)-ALK(20), EML4(13)-ALK(20) | 0.0075 | IHC (positive), RT-PCR (positive) |
| 9 | INSERM | FFPE | Lung biopsy | Positive | Positive | None (positive by 3'/5' imbalance) | 0.0527 | IHC (cytopalasm only), RT-PCR (negative) |
| 10 | Kinki | FFPE | Lung, resection | Positive | Positive | EML4(6a)-ALK(20), EML4(6b)-ALK(20) | 0.3352 | n/a |
| 11 | Kinki | FFPE | Lung, resection | Positive | Positive | EML4(13)-ALK(20) | 0.3767 | n/a |
| 12 | Kinki | Frozen | Pleural fluid | Positive | Positive | EML4(6a)-ALK(20), EML4(6b)-ALK(20) | 0.0033 | n/a |
| 13 | Kinki | Frozen | Pleural fluid | Positive | Positive | EML4(13)-ALK(20) | 0.0907 | n/a |
| 14 | ARUP | FFPE | Lung, lymph node, biopsy | Positive | Positive | EML4(6a)-ALK(20), EML4(6b)-ALK(20) | 0.0451 | n/a |
| 15 | ARUP | FFPE | Not available | Positive | Positive | EML4(13)-ALK(20) | 0.0962 | IHC (positive) |
| 16 | ARUP | FFPE | Lung, lobectomy | Positive | Positive | EML4(13)-ALK(20) | 0.2181 | RT-PCR (positive) |
| 17 | ARUP | FFPE | Lymph node | Positive | Positive | None (positive by 3'/5' imbalance) | 0.0879 | n/a |
| 18 | ARC-Net | FFPE | Lung adenocarcinoma resection | Positive | Negative | n/a | -0.0121 | IHC (weakly positive) |
| 19 | ARC-Net | FFPE | Lung distant metastasis resection | Positive | Positive | HIP1(28)-ALK(20) | -0.0111 | n/a |
| 20 | ARC-Net | FFPE | Lung adenocarcinoma resection | Positive | Positive | EML4(6a)-ALK(20), EML4(6b)-ALK(20) | 0.0019 | n/a |
| 21 | Viollier | FFPE | Lung | Positive | Positive | None (postive by 3'/5' imbalance) | 0.3759 | IHC (positive) |
| 22 | Viollier | FFPE | Lung | Positive | Negative | n/a | -0.0087 | IHC (positive in one lab, negative in one lab) |
| 23 | Viollier | FFPE | Lung | Positive | Positive | EML4(6a)-ALK(20), EML4(6b)-ALK(20) | 0.1646 | IHC (positive) |
| 24 | Viollier | FFPE | Lung | Positive | Negative | n/a | 0.0121 | IHC (positive) |
| 25 | Viollier | FFPE | Lung | Positive | Negative | n/a | 0.0000 | IHC (negative) |
| 26 | Viollier | FFPE | Lung | Positive | Negative | n/a | 0.0099 | IHC (positive) |
| 27 | Radboudumc | FFPE | Lung | Positive | Positive | EML4(6)-ALK(20) | 0.0119 | IHC (positive) |
| 28 | Radboudumc | FFPE | Lung | Positive | Positive | EML4(13)-ALK(20) | 0.2172 | IHC (positive) |
| 29 | CROM | FFPE | Lung, adenosquamous | Positive | Negative | n/a | -0.0431 | IHC (2+) |
| 30 | CROM | FFPE | Lung, adenocarcinoma | Positive | Negative | n/a | 0.0008 | IHC (2+) |
| 31 | CROM | FFPE | Lung, adenocarcinoma with grow pattern signet ring cell | Positive | Negative | n/a | 0.0112 | IHC (negative) |
| 32 | IPATIMUP | FFPE | Bronchoalveolar lavage | Positive | Negative | n/a | 0.0000 | n/a |
| 33 | IPATIMUP | FFPE | Lung adenocarcinoma resection | Positive | Positive | None (positive by 3'/5' imbalance) | 0.3734 | n/a |
| 34 | IPATIMUP | FFPE | Lung adenocarcinoma lymph node metastasis resection | Positive | Positive | EML4(20)-ALK(20) | 0.2166 | n/a |
| 35 | IPATIMUP | FFPE | Lung adenocarcinoma biopsy | Positive | Positive | EML4(14)-ALK(20) | 0.5624 | n/a |
| 36 | Warwick | FFPE | Glandular neoplasia | Positive | Positive | EML4(13)-ALK(20), EML4(20)-ALK(20) | 0.1486 | IHC (positive) |
| 37 | Queen's | FFPE | Lymph node metastatic excision | Positive | Positive | EML4(6)-ALK(20) | 0.3179 | IHC (positive) |
|  |  |  |  |  |  |  |  |  |
| *Negative by ALK FISH* | |  |  |  |  |  |  |  |
| 38 | INSERM | FFPE | Lung | Negative | Negative | n/a | -0.0124 | IHC (negative), RT-PCR (negative) |
| 39 | INSERM | FFPE | Lung | Negative | Negative | n/a | 0.0002 | IHC (negative), RT-PCR (negative) |
| 40 | INSERM | FFPE | Lung | Negative | Negative | n/a | 0.0002 | IHC (negative), RT-PCR (negative) |
| 41 | INSERM | FFPE | Lung | Negative | Negative | n/a | 0.0000 | IHC (negative), RT-PCR (negative) |
| 42 | INSERM | FFPE | Lung | Negative | Negative | n/a | 0.0016 | IHC (negative), RT-PCR (negative) |
| 43 | INSERM | FFPE | Lung | Negative | Negative | n/a | -0.0113 | IHC (negative), RT-PCR (negative) |
| 44 | INSERM | FFPE | Lung | Negative | Negative | n/a | -0.0038 | IHC (negative), RT-PCR (negative) |
| 45 | INSERM | FFPE | Lung | Negative | Negative | n/a | 0.0000 | IHC (negative), RT-PCR (negative) |
| 46 | INSERM | FFPE | Lung | Negative | Negative | n/a | 0.0002 | IHC (negative), RT-PCR (negative) |
| 47 | INSERM | FFPE | Lung biopsy | Negative | Negative | n/a | 0.0094 | IHC (30%), RT-PCR (negative) |
| 48 | INSERM | FFPE | Lung biopsy | Negative | Negative | n/a | 0.0000 | IHC (70%), RT-PCR (negative) |
| 49 | INSERM | FFPE | Lung biopsy | Negative | Negative | n/a | 0.0000 | IHC (20%), RT-PCR (negative) |
| 50 | INSERM | FFPE | Lung biopsy | Negative | Negative | n/a | -0.0225 | IHC (negative), RT-PCR (negative) |
| 51 | INSERM | FFPE | Lung biopsy | Negative | Negative | n/a | 0.0000 | IHC (negative), RT-PCR (negative) |
| 52 | ARUP | FFPE | Lung, FFPE | Negative | Negative | n/a | 0.0012 | n/a |
| 53 | ARC-Net | FFPE | Lung lymph node metastasis resection | Negative | Negative | n/a | 0.0001 | n/a |
| 54 | ARC-Net | FFPE | Lung adenocarcinoma resection | Negative | Negative | n/a | 0.0000 | n/a |
| 55 | ARC-Net | FFPE | Lung adenocarcinoma resection | Negative | Negative | n/a | -0.0025 | n/a |
| 56 | ARC-Net | FFPE | Lung adenocarcinoma resection | Negative | Negative | n/a | -0.0164 | n/a |
| 57 | ARC-Net | FFPE | Lung adenocarcinoma resection | Negative | Negative | n/a | 0.0000 | n/a |
| 58 | ARC-Net | FFPE | Lung adenocarcinoma resection | Negative | Negative | n/a | 0.0001 | n/a |
| 59 | ARC-Net | FFPE | Lung adenocarcinoma resection | Negative | Negative | n/a | -0.0056 | n/a |
| 60 | ARC-Net | FFPE | Lung lymph node metastasis resection | Negative | Negative | n/a | -0.0048 | n/a |
| 61 | ARC-Net | FFPE | Lung adenocarcinoma resection | Negative | Negative | n/a | 0.0000 | n/a |
| 62 | ARC-Net | FFPE | Lung adenocarcinoma resection | Negative | Negative | n/a | 0.0004 | n/a |
| 63 | ARC-Net | FFPE | Lung lymph node metastasis resection | Negative | Negative | n/a | 0.0000 | n/a |
| 64 | ARC-Net | FFPE | Lung adenocarcinoma resection | Negative | Negative | n/a | -0.0072 | n/a |
| 65 | ARC-Net | FFPE | Lung adenocarcinoma resection | Negative | Negative | n/a | -0.0025 | n/a |
| 66 | ARC-Net | FFPE | Lung adenocarcinoma resection | Negative | Negative | n/a | -0.0038 | n/a |
| 67 | ARC-Net | FFPE | Lung lymph node metastasis resection | Negative | Negative | n/a | 0.0000 | n/a |
| 68 | Viollier | FFPE | Lung | Negative | Negative | n/a | 0.0007 | IHC (negative) |
| 69 | Viollier | FFPE | Lung | Negative | Negative | n/a | 0.0006 | n/a |
| 70 | Viollier | FFPE | Lung | Negative | Negative | n/a | 0.0008 | n/a |
| 71 | Viollier | FFPE | Lung | Negative | Negative | n/a | 0.0049 | IHC (negative) |
| 72 | Viollier | FFPE | Lung | Negative | Negative | n/a | 0.0000 | IHC (negative) |
| 73 | Viollier | FFPE | Lung | Negative | Negative | n/a | -0.0059 | IHC (negative) |
| 74 | Viollier | FFPE | Lung | Negative | Negative | n/a | -0.0053 | IHC (negative) |
| 75 | Viollier | FFPE | Lung | Negative | Negative | n/a | 0.0000 | IHC (negative) |
| 76 | Viollier | FFPE | Lung | Negative | Negative | n/a | 0.0000 | IHC (negative) |
| 77 | Viollier | FFPE | Lung | Negative | Negative | n/a | -0.0007 | IHC (negative) |
| 78 | Viollier | FFPE | Lung | Negative | Negative | n/a | 0.0000 | n/a |
| 79 | Viollier | FFPE | Lung | Negative | Negative | n/a | -0.0277 | IHC (negative) |
| 80 | Radboudumc | FFPE | Lung | Negative | Negative | n/a | -0.0003 | IHC (negative) |
| 81 | CROM | FFPE | Lung | Negative | Negative | n/a | 0.0000 | IHC (negative) |
| 82 | CROM | FFPE | Lung | Negative | Negative | n/a | 0.0000 | IHC (negative) |
| 83 | CROM | FFPE | Lung | Negative | Negative | n/a | 0.0000 | IHC (negative) |
| 84 | CROM | FFPE | Lung | Negative | Negative | n/a | 0.0000 | IHC (negative) |
| 85 | CROM | FFPE | Lung | Negative | Negative | n/a | 0.0000 | IHC (negative) |
| 86 | CROM | FFPE | Lung | Negative | Negative | n/a | 0.0000 | IHC (negative) |
| 87 | CROM | FFPE | Lung | Negative | Negative | n/a | 0.0002 | IHC (20%) |
| 88 | CROM | FFPE | Lung | Negative | Negative | n/a | 0.0000 | IHC (negative) |
| 89 | CROM | FFPE | Lung | Negative | Negative | n/a | 0.0000 | IHC (negative) |
| 90 | CROM | FFPE | Lung | Negative | Negative | n/a | 0.0000 | IHC (negative) |
| 91 | CROM | FFPE | Lung | Negative | Negative | n/a | 0.0000 | IHC (negative) |
| 92 | IPATIMUP | FFPE | Lung adenocarcinoma biopsy | Negative | Negative | n/a | 0.0007 | n/a |
| 93 | IPATIMUP | FFPE | Lung adenocarcinoma resection (small fragment) | Negative | Negative | n/a | 0.0013 | n/a |
| 94 | IPATIMUP | FFPE | Lung adenocarcinoma resection (small fragment) | Negative | Negative | n/a | -0.0077 | n/a |
| 95 | IPATIMUP | FFPE | Lung adenocarcinoma resection (small fragment) | Negative | Negative | n/a | -0.0021 | n/a |
| 96 | IPATIMUP | FFPE | Lung adenocarcinoma resection (small fragment) | Negative | Negative | n/a | -0.0012 | n/a |
| 97 | IPATIMUP | FFPE | Lung adenocarcinoma resection (small fragment) | Negative | Negative | n/a | -0.0223 | n/a |
| 98 | IPATIMUP | FFPE | Lung adenocarcinoma resection (small fragment) | Negative | Negative | n/a | 0.0000 | n/a |
| 99 | IPATIMUP | FFPE | Lung adenocarcinoma resection (small fragment) | Negative | Negative | n/a | -0.0095 | n/a |
| 100 | IPATIMUP | FFPE | Lung adenocarcinoma pleura biopsy | Negative | Negative | n/a | -0.0074 | n/a |
| 101 | IPATIMUP | FFPE | Lung adenocarcinoma biopsy | Negative | Negative | n/a | -0.0048 | n/a |
| 102 | Warwick | FFPE | Large cell neuroendocrine | Negative | Negative | n/a | 0.0000 | IHC (negative) |
| 103 | Warwick | FFPE | Undifferentiated large cell | Negative | Negative | n/a | 0.0129 | IHC (negative) |
| 104 | Warwick | FFPE | Undifferentiated large cell | Negative | Negative | n/a | 0.0019 | IHC (negative) |
| 105 | Warwick | FFPE | Glandular neoplasia | Negative | Negative | n/a | 0.0000 | IHC (negative) |
| 106 | Warwick | FFPE | Squamous carcinoma | Negative | Negative | n/a | 0.0000 | IHC (negative) |
| 107 | Queen's | Fresh frozen | Lung resection | Negative | Positive | EML4(6a)-ALK(20) | 0.3179 | IHC (positive) |

| **Table S3. *ROS1* and *RET* Clinical Samples** | | | | | | | |
| --- | --- | --- | --- | --- | --- | --- | --- |
| **Sample** | **Laboratory** | **Sample type** | **Source** | **Reference result (methodology)** | **AmpliSeq result** | **Detected fusion (exon)** | **3'/5' imbalance** |
| *ROS1 samples* | |  |  |  |  |  |  |
| 10^*†^ | Kinki | FFPE | Lung, resection | Negative (mass spectrometry) | Negative | n/a | -1.7843 |
| 11^*†^ | Kinki | FFPE | Lung, resection | Negative (mass spectrometry) | Negative | n/a | -0.4617 |
| 12^*†^ | Kinki | Frozen | Pleural fluid | Negative (mass spectrometry) | Negative | n/a | -0.0038 |
| 13^*†^ | Kinki | Frozen | Pleural fluid | Negative (mass spectrometry) | Negative | n/a | -0.0279 |
| 23^*^ | Viollier | FFPE | Lung | Negative (FISH) | Negative | n/a | -0.3519 |
| 68^*^ | Viollier | FFPE | Lung | Negative (FISH) | Negative | n/a | -0.0123 |
| 71^*^ | Viollier | FFPE | Lung | Positive (FISH) | Negative | n/a | -0.2300 |
| 72^*†^ | Viollier | FFPE | Lung | Positive (FISH) | Positive | CD74(6)-ROS1(34) | 1.0068 |
| 73^*^ | Viollier | FFPE | Lung | Negative (FISH) | Negative | n/a | -0.0216 |
| 74^*†^ | Viollier | FFPE | Lung | Negative (FISH) | Negative | n/a | -0.0138 |
| 75^a^ | Viollier | FFPE | Lung | Negative (FISH) | Negative | n/a | -0.0197 |
| 76^*†^ | Viollier | FFPE | Lung | Negative (FISH) | Negative | n/a | -0.0326 |
| 77^*†^ | Viollier | FFPE | Lung | Negative (FISH) | Negative | n/a | -0.0037 |
| 108^†^ | Kinki | FFPE | Lung, resection | Negative (mass spectrometry) | Negative | n/a | -0.1727 |
| 109^†^ | Kinki | FFPE | Lung, resection | Negative (mass spectrometry) | Negative | n/a | -0.0017 |
| 110^†^ | Kinki | FFPE | Lung, resection | Negative (mass spectrometry) | Negative | n/a | -0.6244 |
| 111^†^ | Kinki | FFPE | Lung, resection | Negative (mass spectrometry) | Negative | n/a | -0.0039 |
| 112^†^ | Kinki | FFPE | Lung, resection | Negative (mass spectrometry) | Negative | n/a | -0.1248 |
| 113^†^ | Kinki | FFPE | Lung, resection | Negative (mass spectrometry) | Negative | n/a | -0.1335 |
| 114 | ARUP | FFPE | Lung | Positive (IHC) | Positive | EZR(10)-ROS1(34), EZR(10)-ROS1(35) | 0.1874 |
| 115 | ARUP | FFPE | Lung | Positive (IHC) | Positive | EZR(10)-ROS1(34), EZR(10)-ROS1(33) | -0.0791 |
| 116 | ARUP | FFPE | Lung | Negative (IHC) | Negative | n/a | -0.3560 |
|  |  |  |  |  |  |  |  |
| *RET samples* | |  |  |  |  |  |  |
| 10^*†^ | Kinki | FFPE | Resection | Negative (mass spectrometry) | Negative | n/a | 0.0211 |
| 11^*†^ | Kinki | FFPE | Resection | Negative (mass spectrometry) | Negative | n/a | 0.0023 |
| 12^*†^ | Kinki | Frozen | Pleural fluid | Negative (mass spectrometry) | Negative | n/a | -0.0026 |
| 13^*†^ | Kinki | Frozen | Pleural fluid | Negative (mass spectrometry) | Negative | n/a | -0.0029 |
| 72^*†^ | Viollier | FFPE | Lung | Negative (FISH) | Negative | n/a | -0.0033 |
| 74^*†^ | Viollier | FFPE | Lung | Negative (FISH) | Negative | n/a | 0.0026 |
| 76^*^ | Viollier | FFPE | Lung | Negative (FISH) | Negative | n/a | 0.0050 |
| 77* | Viollier | FFPE | Lung | Negative (FISH) | Negative | n/a | 0.0009 |
| 108^†^ | Kinki | FFPE | Lung, resection | Negative (mass spectrometry) | Negative | n/a | 0.0014 |
| 109^†^ | Kinki | FFPE | Lung, resection | Negative (mass spectrometry) | Negative | n/a | 0.0004 |
| 110^†^ | Kinki | FFPE | Lung, resection | Negative (mass spectrometry) | Negative | n/a | 0.0010 |
| 111^†^ | Kinki | FFPE | Lung, resection | Negative (mass spectrometry) | Positive | None (positive by 3'/5' imbalance) | 0.1970 |
| 112^†^ | Kinki | FFPE | Lung, resection | Negative (mass spectrometry) | Negative | n/a | -0.0009 |
| 113^†^ | Kinki | FFPE | Lung, resection | Negative (mass spectrometry) | Negative | n/a | 0.0009 |
| 117 | Kinki | FFPE | Lung, biopsy | Positive (mass spectrometry, RT-PCR) | Positive | CCDC6(12)-RET(1) | 0.2293 |
| ^*^Samples that were also tested for ALK fusions (see Table S2). | | | | |  |  |  |
| ^†^Samples previously tested for both ROS1 and RET fusions. | | | | |  |  |  |
